# Interocular Difference of Peripheral Refraction in Anisomyopic Eyes of Schoolchildren

**DOI:** 10.1371/journal.pone.0149110

**Published:** 2016-02-16

**Authors:** Junhong Chen, Ji C. He, Yunyun Chen, Jingjing Xu, Haoran Wu, Feifu Wang, Fan Lu, Jun Jiang

**Affiliations:** 1 School of Optometry & Ophthalmology, Wenzhou Medical College, Wenzhou, Zhejiang, China; 2 New England College of Optometry, Boston, Massachusetts, United States of America; LV Prasad Eye Institute, INDIA

## Abstract

**Purpose:**

Refraction in the peripheral visual field is believed to play an important role in the development of myopia. The purpose of this study was to investigate the differences in peripheral refraction among anisomyopia, isomyopia, and isoemmetropia for schoolchildren.

**Methods:**

Thirty-eight anisomyopic children were recruited and divided into two groups: (1) both eyes were myopic (anisomyopic group, AM group) and (2) one eye was myopic and the contralateral eye was emmetropic (emmetropic anisomyopic group, EAM group). As controls, 45 isomyopic and isoemmetropic children were also recruited with age and central spherical equivalent (SE) matched to those of the AM and EAM groups. The controls were divided into three groups: (1) intermediate myopia group (SE matched to the more myopic eye of AM group), (2) low myopia group (SE matched to the less myopic eye of AM group and the more myopic eye of EAM group), and (3) emmetropia group (SE matched to the less myopic eye of EAM group). Peripheral refraction at 7 points across the central ±30° on the horizontal visual field with a 10° interval was measured with an autorefractor. Axial length (AL), corneal curvature (CC), and anterior chamber depth (ACD) were also determined by using the Zeiss IOL-Master.

**Results:**

The relative peripheral spherical equivalent [RPR(M)] and relative peripheral spherical value [RPR(S)] of the more myopic eye was shifted more hyperopically than the contralateral eye in both the AM and the EAM groups (both p<0.0001). The RPR(M, S) of the less myopic eyes in the AM and EAM groups showed a relatively flat trend across the visual field and were not significantly different from the emmetropia group. The RPR(M, S) of less myopic eyes in the AM group were shifted less hyperopically than in the isomyopic low myopia group and the more myopic eye of the EAM group [RPR(M), p = 0.007; RPR(S), p = 0.001], although the central SEs of the three groups were not significantly different from each other. However, RPR(M, S) of the more myopic eyes were not different from the corresponding isomyopic groups. There was also no significant difference in the relative peripheral astigmatism [RPR(J0, J45)] between the more and the less myopic eyes in either the AM or the EAM group.

**Conclusion:**

Refraction of anisomyopia differs between the two eyes not only at the central visual field but also at the off-axis periphery. The relative peripheral refraction of the more myopic eye of anisomyopia was shifted hyperopically, as occurs in isomyopia with similar central subjective SE values. Less myopic eyes were much less hyperopically shifted in relative peripheral refraction than the corresponding isomyopic eyes, but are comparable to emmetropic eyes. This emmetropia-like relative peripheral refraction in less myopic eyes might be a factor responsible for slowing down the progression of myopia.

## Introduction

Anisomyopia is defined as a difference of more than one diopter (D) in refractive status between the two eyes of a myopic individual. Babies are usually hyperopic and have similar interocular refractive power in both eyes [1, 2]. The incidence of anisomyopia is low in young children [2–4] but increases to 6.7%-7.7% by 30 years of age [5, 6]; however, the mechanism by which it develops is still unresolved. Because both eyes of the anisomyopic individual share the identical genetic base and also the same visual environment, it becomes very difficult to identify the factor(s) that is/are responsible for the differential development in refractive power between the two eyes. A complete understanding of the mechanism underlying this development is therefore important not only for finding effective interventions to its development but also for providing insight to myopia research in general.

A variety of optical and bio-mechanical factors that are believed to be involved in myopia development for isomyopes have been previously examined to test their influence on the development of anisomyopia [7]. However from the experimental observations, no single factor has been consistently found to be different between the two eyes. For example, wavefront aberration of the eye is a factor contributing to retinal image blurring and was suggested to be involved in myopia development, but anisomyopia studies showed only very slight or non-significant interocular difference in anisometropes [8–10]. Intraocular pressure (IOP) was also suggested to be a factor contributing to myopia development, but in several recent studies, no significant interocular difference in IOP was found between the anisometropic fellow eyes [10–13]. Thus the mechanism underlying asymmetrical development between the anisomyopic eyes remains elusive.

Recently, increasing interest has been showed in the area of peripheral visual field refraction since peripheral refraction is believed to play an important role in the development of myopia.

According to an early study, emmetropic eyes with hyperopic refraction in the peripheral field relative to the fovea were associated with myopic development over time [14]. Further studies [15–17] have demonstrated that myopes tend to have a relative hyperopic refraction at the periphery, while emmetropes exhibited a relative peripheral myopic refractive error. Animal studies provided further evidence to support the hypothesis that peripheral refraction influences refractive development [18–20].

Although a variety of optical factors have been previously examined for their influence on the development of anisomyopia, the role of peripheral refraction has not been explored. Investigation on involvement of the peripheral refraction in anisomyopia development is important not only for revealing underlying mechanism of the development of anisomyopia but also for using effective clinical treatment to provent anisomyopia development in children. This study was designed to investigate the possible contribution of peripheral refraction to anisomyopia development by measuring peripheral refraction for a group of schoolchildren with anisomyopia and also a group of isometropic children with central spherical equivalent matching anisomyopia for comparison.

## Methods

### Subjects

This study was approved by the Ethics Committee of Wenzhou Medical University, Wenzhou, China. All subjects were treated in accordance with the tenets of the Declaration of Helsinki. Written consent was obtained from the parents or guardians of the included participants after a detailed explanation of the procedures, discomfort and the side effect of the topical cycloplegic used.

Thirty-eight anisomyopic schoolchildren with a difference in spherical equivalent (SE) between the two eyes greater than 1.00 D were recruited from the Eye Hospital of Wenzhou Medical University. All children were 11–15 years old (Table 1) and were given a full ocular examination, including binocular vision, objective and subjective refraction, and anterior segment and fundus inspection. Participants were divided into two groups according to their subjective refractive error status. Those for which both eyes were myopic were designated the AM group. Those in which one eye was myopic and the contralateral eye was emmetropic were designated the EAM group. The difference in SE values between the eyes was 2.36±1.24 D for the AM group and 1.66±0.47 D for the EAM group.

**Table 1:** Subject number, age profile, mean subjective spherical error, cylinder, and SE of anisomyopic, isomyopic, and isoemmetropic children.

	AM group			EAM group			Isometropic groups		
	MM	LM	*P*	MY	EM	*P*	Intermediate myopia group	Low myopia group	Emmetropia group
Gender									
Male	6			10			7	6	8
Female	9			13			10	9	5
Total	15			23			17	15	13
Age range (y)	11–15			11–15			11–15	11–14	11–15
Mean age (y)	12.6±1.5			13.0±1.3			12.4±1.3	12.1±0.9	12.2±1.2
Spherical component (D)	-3.78±1.51	-1.33±1.02	<0.0001	-1.50±0.41	0.20±0.28	<0.0001	-3.74±0.31	-1.47±0.23	0.26±0.37
Cylindrical component (D)	-0.20±0.32	-0.38±0.30	0.151	-0.15±0.24	-0.23±0.34	0.397	-0.26±0.36	-0.17±0.26	-0.26±0.30
SE value (D)	-3.88±1.49	-1.53±1.06	<0.0001	-1.58±0.42	0.08±0.24	<0.0001	-3.88±0.28	-1.55±0.24	0.13±0.37

AM, anisomyopic with both eyes myopic; EAM, anisomyopic with one eye emmetropic; Intermediate myopia group with a subjective central SE similar to the more myopic eye (MM) of AM group; Low myopia group with a subjective central SE that was similar to the less myopic eye (LM) of the AM group and the myopic eye (MY) of EAM group; Emmetropia group with a subjective central SE that was similar to the emmetropic eye (EM) of EAM group.

A control group of 45 isomyopic and isoemmetropic children for whom the difference of SE between the two eyes was <0.50 D was also recruited. The ages and central SEs of this group were matched to those of the AM and EAM groups. Among these participants, we designated three sub-groups (Table 1): (1) the intermediate myopia group had a subjective central SE that was similar to the more myopic eye of the AM group, (2) the low myopia group had a subjective central SE that was similar to the less myopic eye of the AM group and the myopic eye of EAM group, and (3) the emmetropic group had a subjective central SE that was similar to the emmetropic eye of EAM group. Emmetropia is defined as the central subjective spherical equivalent value between -0.25D~+0.50D. There were no statistically significant differences between any two groups with respect to age.

Subjects with best-corrected visual acuity worse than 20/20, astigmatism more than 1.00 D, strabismus, a history of rigid contact lens wear within 6 months, ocular surgery, trauma, or other ocular or systemic disease were excluded from the study.

There were no significant differences in the refractive components among the less myopic eyes of the AM group, isomyopic low myopia group, and the myopic eyes of the EAM group (0.648<p<0.964). Additionally, the refractive components in the anisomyopic groups were not significantly different from the corresponding isometropic groups (independent-samples t-test, p>0.05 for the more myopic eyes in the AM group vs. the intermediate myopia group; independent-samples t-test, p>0.05 for emmetropic eyes in the EAM group vs. the emmetropia group).

### Instrumentation

Central and peripheral refractive errors across the central 60° horizontal field were measured by an open-field, infrared autorefractor (Grand Seiko Model WAM-5500, Hiroshima, Japan) as previously described [15, 21, 22]. Axial length (AL), corneal curvature (CC) over a 2.30 mm diameter of the central anterior corneal surface, and anterior chamber depth (ACD) of the eye were measured using the IOL-Master (Carl Zeiss Meditec, Jena, Germany).

### Procedure

Except for subjective refraction, other parameters were assessed after cycloplegia was induced by 1% cyclopentolate hydrochloride eyedrops delivered twice, 5 minutes apart. When peripheral refraction was measured, a mobile penlight mounted on an arc iron shelf (33-cm radius) served as a target to change the visual axis from the measurement axis of the autorefractor. The center of arc iron shelf was set to be coincident with the center of rotation of the eye. The penlight was moved at 10° intervals through ±30° in the horizontal visual field. Each subject was asked to move his/her eye toward the fixation target while keeping the head firmly against the forehead and chin rests. During the measurements, the fellow eye was occluded and the instrument mark was maintained to be coincident over the pupil center of the test eye on the autorefractor’s screen. We used the central pupil alignment method to ensure the reliability of the results [23]. The measurement was repeated 4 times at each fixation position, and the average value was calculated for data analysis.

After the measurement of peripheral refraction, the ACD, CC, and AL were determined for subjects in the anisomyopic group with the IOL Master. With the forehead and chin resting on the instrument, each subject was instructed to blink his/her eye before the measurement was taken to maintain the integrity of the tear film. The measurement was repeated 5 times for AL and 3 times for CC and ACD. Measurements were taken by the same operator and using the same instrument to minimize potential variability of measurements.

## Data Analysis

The central and peripheral refractive error readings were obtained as the sphere (S), cylinder (C), and axis (θ), and the measurements were then converted to power vectors by using following equations [24]:

$$\begin{array}{l} \text{M = S + C/2,}\\ \text{J0 = -C cos(2}\theta \text{)/2,}\\ \text{J45 = -C sin(2}\theta \text{)/2,} \end{array}$$

where M was the spherical equivalent, S was the spherical value, J0 and J45 were the power of the two Jackson cross-cylinder components. Relative peripheral refraction (RPR) was defined as the difference in refractive error between the peripheral refractive value and the central refractive value.

Statistical analyses were performed using SPSS 16.0 (SPSS Inc., Chicago, IL, USA). Peripheral refractive values were compared using repeated-measures analysis of variance (ANOVA). For statistically significant differences, paired-sample t-tests, independent-samples t-tests, and one-way ANOVAs were applied to analyze the data at each peripheral position. Least-significant difference (LSD) correction was employed to adjust the critical values. Paired-sample t-tests were also performed for interocular comparisons of AL, CC, and ACD.

## Results

### Peripheral Refraction of Anisomyopic Groups

Mean peripheral refractions (PR) were measured for the more and less myopic eyes in both the AM and EAM groups (S1 Fig) with the spherical equivalents M, J0, and J45. The profile of the PR(M) in the more myopic eye was significantly more myopic at all locations than that in the less myopic eye for both the AM and the EAM groups (S1 Fig). However, the profiles of astigmatism components PR(J0) (S1 Fig) and PR(J45) (S1 Fig) were very similar to each other for the two eyes of each group.

To determine the relative peripheral refraction (RPR) for both the AM and EAM groups, we normalized the peripheral refraction to the central refraction. The profile of the relative peripheral spherical equivalent [RPR(M)] and the relative peripheral spherical value [RPR(S)] in the more myopic eye (S2 Fig) exhibited a hyperopic shift. In contrast, the profile in the less myopic eye was relatively flat for both the AM and the EAM groups. There were significant differences in the RPR(M, S) between the anisomyopic eyes in both the AM and EAM groups [RPR(M, S) *p*<0.0001 for both groups]. For the AM group, the differences in RPR(S) were significant at all angles; however, the differences in RPR(M) were significant at all angles except at the temporal 10° visual field. For the EAM group, the interocular differences for RPR(M, S) were present only at 20° and 30° in both at temporal and nasal visual fields (Table 2).

**Table 2:** Paired-sample p-values of all field angles for RPR(M, S) between the more myopic eye and the less myopic eye in AM and EAM groups.

Vectors	Groups	Field angles					
		T30°	T20°	T10°	N10°	N20°	N30°
RPR(M)	AM	0.002	0.002	0.139	0.017	0.005	0.002
	EAM	<0.0001	0.003	0.266	0.139	<0.0001	<0.0001
RPR(S)	AM	<0.0001	<0.0001	0.041	0.019	0.004	0.001
	EAM	<0.0001	<0.0001	0.286	0.057	<0.0001	<0.0001

T, temporal; N, nasal; RPR(M), relative peripheral spherical equivalent; RPR(S), relative peripheral spherical value; AM, both eyes myopic; EAM, one eye was myopic and the contralateral eye was emmetropic.

For both the AM and EAM groups, there was a myopic shift in RPR(J0) values. The shift increased with the visual angle, but the changes between the two eyes were not significant (AM: p = 0.536; EAM: p = 0.419). There were small linear changes in the RPR(J45) for both eyes in the AM and EAM groups, but these changes were also not significant (AM: p = 0.681; EAM: p = 0.255).

### Peripheral Refraction of the Isometropic Groups

For the intermediate and low myopia groups, the myopic shift in the profiles of the PR(M) were greater than that of the emmetropia group (S3 Fig). However, the profiles of astigmatism components PR(J0) (S3 Fig) and PR(J45) (S3 Fig) were similar to each other among the three groups.

While there was a hyperopic shift in the RPR(M) (S4 Fig) and RPR(S) (S4 Fig) profiles of the intermediate and low myopia groups, there was none in the emmetropia group (S4 Fig). There was a greater hypermetropic shift in the RPR(M, S) profiles in intermediate myopia group than the low myopia group, but only the shift in RPR(M) reached statistical significance (p = 0.012). There were no significant differences in RPR(J0) (p = 0.525, S4 Fig) or RPR(J45) (p = 0.315, S4 Fig) among the three groups.

### Comparison of Peripheral Refraction between the Anisomyopic Groups and the Isometropic Group

We compared the relative peripheral refraction among the anisomyopic and the isometropic groups. The RPR(M, S) were significantly different among the less myopic eyes of the AM group, isomyopic low myopia group, and the myopic eye of the EAM group [RPR(M), p = 0.007; RPR(S), p = 0.001], even though the three groups had almost the same degree of central myopia (Table 1). The RPR(M, S) in the isomyopic low myopia group and the myopic eye of the EAM group had a greater hypermetropic shift than did the less myopic eye of the AM group. The differences were more obvious at the 30° visual fields for RPR(M) (T30, p = 0.022; N30, p = 0.013; One-way ANOVA) and at the 20° and 30° visual fields for RPR(S) (T30, p = 0.002; T20, p = 0.017; N20, p = 0.032; N30, p = 0.02; One-way ANOVA). Multiple comparisons showed that the RPR(M, S) were significantly different between the less myopic eye of the AM group and the isomyopic low myopia group (RPR(M), 0.032<p<0.026; RPR(S), 0.014< p<0.005; LSD correction applied) and the less myopic eye of the AM group and the myopic eye of the EAM group (RPR(M), 0.008<p<0.003; RPR(S), 0.009< p<0.0001; LSD correction applied). However for RPR(M, S), there were no differences at any angle between the myopic eye of the EAM group and corresponding isomyopic low myopia group (Table 3). Similarly, there were no differences in the RPR(M, S) values of the more myopic eyes of the AM group compared to the corresponding isomyopic intermediate myopia group [RPR(M), p = 0.78; RPR(S), p = 0.085].

**Table 3:** P-values for multiple comparisons (LSD correction applied) of all field angles for RPR(M, S) among the less myopic eye of AM group, the low myopia group and the more myopic eye of the EAM group.

Vectors	Groups	Field angles					
		T30°	T20°	T10°	N10°	N20°	N30°
RPR(M)	AM group & Low myopia group	0.026	0.083	0.854	0.351	0.25	0.032
	AM group & EAM group	0.008	0.098	0.231	0.118	0.027	0.003
	EAM group & Low myopia group	0.487	0.999	0.220	0.411	0.169	0.261
RPR(S)	AM group & Low myopia group	0.005	0.014	0.363	0.165	0.119	0.01
	AM group & EAM group	<0.0001	0.007	0.061	0.092	0.009	0.001
	EAM group & Low myopia group	0.259	0.652	0.222	0.655	0.159	0.177

T, temporal; N, nasal; RPR(M), relative peripheral spherical equivalent; RPR(S), relative peripheral spherical value; AM, both eyes myopic; EAM, one eye was myopic and the contralateral eye was emmetropic

While the less myopic eye of the AM group and the emmetropic eye of the EAM group had a flat profile (S2 Fig), there were no significant differences in RPR(M, S) among the less myopic eyes of the AM group, the emmetropic eyes of the EAM group, and the eyes of the isoemmetropia group [RPR(M), p = 0.539; RPR(S), p = 0.691]. For RPR(J0) (p = 0.333) and RPR(J45) (p = 0.473), there were no significant differences among the less myopic eyes of the AM group, the isomyopic eyes of the low myopia group, and the myopic eyes of the EAM group.

### Axial Length, Corneal Curvature, and Anterior Chamber Depth in the Anisomyopic Groups

The AL, CC, and ACD were measured for the more myopic and less myopic eyes of both the AM and EAM groups (Table 4). The AL difference between the eyes was 0.96±0.56 mm in AM group and 0.66±0.31 mm in EAM group (Table 5). There were statistically significant differences in AL between the more myopic eye and the less myopic eye in anisomyopic groups (both *p*<0.0001). For the CC and ACD, there were no differences between anisomyopic eyes in either group.

**Table 4:** AL, ACD, and CC value for the more myopic eye and the less myopic eye of the two anisomyopic groups.

	AM group			EAM group		
Ocular Component	MM	LM	*P*	MY	EM	*P*
AL (mm)	25.25±1.08	24.30±0.91	<0.0001	24.46±0.82	23.80±0.80	<0.0001
ACD (mm)	3.69±0.32	3.68±0.27	0.468	3.66±0.18	3.63±0.18	0.192
Flattest meridian of CC (mm)	7.84±0.27	7.85±0.27	0.346	7.86±0.21	7.88±0.22	0.327
Steepest meridian of CC (mm)	7.65±0.31	7.63±0.32	0.109	7.68±0.20	7.66±0.23	0.131

AM, both eyes myopic; EAM, one eye was myopic and the contralateral eye was emmetropic; MM, more myopic; LM, less myopic, MY, myopic, EM, emmetropic, AL, axial length; ACD, anterior chamber depth; CC, cornea curvature.

**Table 5:** Differences of AL, CC, and ACD between the more myopic eye and the less myopic eye in the anisomyopic groups.

	AM group	EAM group
ΔAL (mm)	0.96±0.56	0.66±0.31
ΔACD (mm)	0.02±0.09	0.03±0.11
ΔFM (mm)	-0.01±0.06	-0.01±0.05
ΔSM (mm)	0.03±0.06	0.02±0.07

AM, both eyes myopic; EAM, one eye was myopic and the contralateral eye was emmetropic; Δ, difference between the more myopic eye and the less myopic eye; AL, axial length; ACD, anterior chamber depth; FM, flattest corneal meridian; SM, steepest corneal meridian.

## Discussion

By definition, anisomyopes have different central refractive powers between the fellow eyes. Due to the difference of central refraction, it is reasonable to predict that anisomyopes could have different relative peripheral refractions between the two eyes because even in isomyopic eyes the relative peripheral refraction is different when the central refraction was changed [22, 25]. By measuring peripheral refraction in schoolchildren in the current study, we found that the anisomyopic children had different relative peripheral refraction between the fellow eyes. However, the difference in relative peripheral refraction was much greater than would be predicted from the corresponding isomyopic eyes.

The relative peripheral refraction in the more myopic eye of anisomyopic eyes was very comparable to that in isomyopic eyes with similar central subjective refraction; however, it was much different in the less myopic eye of anisomyopic eyes from the isomyopic eyes with similar central refraction. This was clearly evident when the RPR(M, S) were compared among the nisoamyopic and the isometropic groups. The RPR(M, S) in the less myopic eyes of the AM group was significantly different in the isomyopic eyes of the low myopia group, even though the mean central subjective-refractions were about the same for the two groups. The RPR(M, S) in the less myopic eyes of the AM group were much less hyperopic at all locations than in the isomyopic low myopia group, but the differences were significant only at T and N30° for RPR(M, S) and at T20° for RPR(S). The relative peripheral refraction in the more myopic eyes of the AM group showed the hyperopically shifted which was similar to the corresponding isomyopic eyes. It seemed reasonable to suppose that the relative peripheral refraction in the less myopic eyes of the AM group was similar to the corresponding isomyopic eyes. In contrast, the less myopic eyes had a relative peripheral refraction that was very similar to that in the emmetropic eyes, which was relatively flat across the visual field, as reported for isoemmetropic eyes [15, 16, 25]. There was no difference in the peripheral refraction between the less myopic eye of the AM group and the isoemmetropia group. Thus the more myopic eyes of anisomyopes have relative peripheral refractions that are very similar to isomyopic eyes. The relative peripheral refractions of less myopic eyes are very different from the isomyopic eyes even when the central refraction is similar and very close to isoemmetropic eyes.

We also found that the myopic eye of the EAM group had similar relative peripheral refraction to the isomyopic low myopia group at all visual fields. Because the differences in RPR(M, S) between the less myopic eye of the AM group and the isomyopic low myopia group were significant, it is not surprising that the peripheral refraction between the less myopic eye of the AM group and the myopic eye of the EAM group was significant even though the three groups had similar central subjective-refractions.

The relative peripheral astigmatism components J0 and J45 were not significant between the fellow eyes for anisomyopes. Thus the differences in relative peripheral refraction between the anisomyopic eyes was due to spherical error alone, with no contribution by astigmatism. The peripheral spherical error is associated with the retinal shape profile [26], and this may explain the differences in peripheral refraction between the two anisomyopic eyes.

The AL, CC, and ACD were measured under cycloplegia, which had no significant effect on AL or CC, but it did increase ACD as shown by others[27, 28]. However, cycloplegia could not have impacted the overall results of this study because we compared the ocular dimensions between the anisomyopic eyes of each subject. In both groups of anisomyopes, the AL of the more myopic eye was significantly greater than that of the less myopic eye, which is consistent with previous reports[10, 29–31]. Vincent et al. used the E300 videokeratoscope to measure corneal topography of anisomyopic eyes [10]. They found that the more myopic eyes were slightly more powerful in the flattest and steepest corneal meridians. In contrast, using the IOL-Master to measure the corneal meridians, we did not find any differences in corneal meridians between the anisometropic eyes. Using the Pentacam, Yuksel et al. reported no differences of CC or ACD between the hyperopic anisometropic eyes of amblyopia patients [32]. We also found no difference of the ACD between the more myopic eye and the less myopic eye. This finding is different from that by Kuo et al [29]. They used optical coherence tomography A-scans to measure the ACD in anisometropes and reported that the mean ACD in the more myopic eye was deeper than that in the other eye. Even though the difference in ACD that we measured was not statistically significant, it tended to be similar to that reported by Kuo et al. The lack of statistical significance in our study population could be due to the difference in refractive power of the subjects. In the study by Kuo et al., the difference of mean SE between anisometropic eyes, 4.87 D, which was more severe than ours, 1.52 D.

How anisomyopia develops is still an open question for myopia research. Premature birth and defects in optical components of the eye, such as unilateral cataracts, pseudophakia, and vitreous hemorrhage can contribute to asymmetric eye development [5, 31, 33–35]. Genetic inheritance is also highly associated with significant differences in refraction between the eyes [36–38]. However except for refractive errors, the subjects enrolled in this study had very normal eyes, thus the causes of their anisometropia are not likely to be the result of these factors and/or mechanisms.

Previous studies have been conducted to explore the optical and mechanical factors of anisometropia, but no consistent results have reported [9–13, 32, 39, 40]. Our finding of emmetropia-like peripheral refraction in the less myopic eye suggests that peripheral refraction plays a role in the development of anisomyopia. The development of the eye with less myopia could be due to a smaller hyperopic shift in relative peripheral refraction compared to isomyopic eyes with a similar central refraction. However, if the relative peripheral refraction in the less myopic eye in anisomyopic subjects is like the isoemmetropic eye in our study, then it might be reasonable to argue that the eye should not develop myopia, as occurs in the development of isoemmetropic eyes. Thus the question remains as to what causes the less myopic eye in the AM group to develop myopia. At the moment, our results do not provide an answer for that question. One possibility is that the development of anisomyopia is the result of the interaction of multiple factors rather than being due to only the relative peripheral refraction. The effect of emmetropia-like peripheral refraction in the less myopic eye could be to slow down the progression of myopia but not to prevent the development of it.

Currently, peripheral refraction is thought to be a factor that contributes to myopia development and progression, though some longitudinal studies have reported baseline peripheral refraction did not predict the onset or progression of myopia[22, 41]. Orthokeratology (OK) and specially designed soft contact lenses can reduce the rate of progression of childhood myopia [42–45]. Some studies reported that OK significantly reduced central myopia and converted relative peripheral refraction to relative peripheral myopia at the same time [46, 47]. Further, soft contact lenses designed to reduce relative peripheral hyperopia have been shown to decrease the rate of myopia progression [48]. However, the underlying mechanism by which peripheral refraction affects central refraction is still not well understood. The same situation applies to our cross-sectional study. We were unable to develop an understanding of the mechanism by which peripheral refraction affects the development of anisomyopia. A relevant question is why the relative refraction of low myopic eyes is different from the isomyopic eyes and the more myopic eyes even though they have similar central refractions. This is a difficult question and will require further study.

In clinical practices, significant anisometropia is a predictor of poor binocular function[49]. The poor binocular function could be due to not only the refractive difference between the two eyes at the central visual field but the peripheral field also. Thus, special attention on difference in peripheral refraction might be required when visual correction for anisomyopia is considered.

Effective methods are needed to prevent interocular difference of refractive error from developing. In a recent study, an 11-year-old anisomyopic boy was reported fitting with an ortho-k lens in left eye to slow down myopia progression, and it was found that the ortho-k lens effectively decreased the amount of anisomyopia[50]. Because the ortho-k lens was suggested to be effective to correct peripheral hyperopia[46, 47], the effectivity of the ortho-k treatment could be contributed from the correction of the peripheral refraction also. Therefore, methods with capability of correcting both central and peripheral refractive errors are expected to be more effective to treat anisomyopia and probably to retard progression of anisomyopia.

In summary, by measuring peripheral refraction in anisomyopic schoolchildren, we found that in addition to differences in central field refraction between the two eyes, there were also significant differences in peripheral refraction. The more myopic eye of anisomyopia had a hyperopic shift in relative peripheral refraction that was similar to the shift in corresponding isomyopic eyes. The less myopic eye of anisomyopia had a much smaller hyperopic shift that corresponded with that in isomyopic eyes with similar central SE values. Further, the relative peripheral refraction was comparable to that of emmetropic eyes. This emmetropia-like relative peripheral refraction in the less myopic eye might be a factor responsible for slowing down the progressive development of myopia.

## Supporting Information

S1 Fig Mean peripheral refraction of anisomyopic children. (DOCX)

S2 Fig Mean relative peripheral refraction of anisomyopic children. (DOCX)

S3 Fig Mean peripheral refraction of isometropic children. (DOCX)

S4 Fig Mean relative peripheral refraction of isometropic children. (DOCX)
