# Supplementary material for: Interocular Difference of Peripheral Refraction in Anisomyopic Eyes of Schoolchildren
Source: PLoS One. 2016 Feb 16;11(2):e0149110. doi: 10.1371/journal.pone.0149110 (PMC4755577; doi:10.1371/journal.pone.0149110)
Supplement: S1 Fig — (DOCX) [file pone.0149110.s001.docx]

Figure S1 Peripheral spherical equivalent (a), peripheral astigmatism component J0 (b), and peripheral astigmatism component J45 (c) of the more myopic and less myopic eyes from the anisomyopic (AM) and emmetropic anisomyopic (EAM) groups. PR(M), peripheral spherical equivalent; PR(J0), power of peripheral astigmatism Jackson cross-cylinder component J0; PR(J45), power of peripheral astigmatism Jackson cross-cylinder component J45; D, diopter; T, temporal visual field; N, nasal visual field; Error bar represents one standard error of the mean.
