# Supplementary material for: Interocular Difference of Peripheral Refraction in Anisomyopic Eyes of Schoolchildren
Source: PLoS One. 2016 Feb 16;11(2):e0149110. doi: 10.1371/journal.pone.0149110 (PMC4755577; doi:10.1371/journal.pone.0149110)
Supplement: S2 Fig — (DOCX) [file pone.0149110.s002.docx]

Figure S2 Relative peripheral spherical equivalent (a), relative peripheral spherical value (b), relative peripheral astigmatism component J0 (c), and relative peripheral astigmatism component J45 (d) of the more myopic and less myopic eyes from the anisomyopic groups and the emmetropic anisomyopic group. RPR(M), relative peripheral spherical equivalent; RPR(S), relative peripheral spherical value; RPR(J0), relative power of peripheral astigmatism Jackson cross-cylinder component J0; RPR(J45), relative power of peripheral astigmatism Jackson cross-cylinder component J45; D, diopter; T, temporal visual field; N, nasal visual field; Error bar represents one standard error of the mean.
