# Supplementary material for: Interocular Difference of Peripheral Refraction in Anisomyopic Eyes of Schoolchildren
Source: PLoS One. 2016 Feb 16;11(2):e0149110. doi: 10.1371/journal.pone.0149110 (PMC4755577; doi:10.1371/journal.pone.0149110)
Supplement: S3 Fig — (DOCX) [file pone.0149110.s003.docx]

Figure S3 Mean peripheral spherical equivalent (a) and peripheral astigmatism components J0 (b) and J45 (c) of the right eyes from the two isomyopic groups and the emmetropic group. PR(M), peripheral spherical equivalent; PR(J0), power of peripheral astigmatism Jackson cross-cylinder component J0; PR(J45), power of peripheral astigmatism Jackson cross-cylinder component J45; D, diopter; T, temporal visual field; N, nasal visual field; Error bar represents one standard error of the mean.
